# Predict Ki-67 Positive Cells in H&E-Stained Images Using Deep Learning Independently From IHC-Stained Images

**DOI:** 10.3389/fmolb.2020.00183

**Published:** 2020-08-04

**Authors:** Yiqing Liu, Xi Li, Aiping Zheng, Xihan Zhu, Shuting Liu, Mengying Hu, Qianjiang Luo, Huina Liao, Mubiao Liu, Yonghong He, Yupeng Chen

**Affiliations:** ^1^Department of Life and Health, Tsinghua Shenzhen International Graduate School, Shenzhen, China; ^2^Department of Gastroenterology, Peking University Shenzhen Hospital, Shenzhen, China; ^3^Department of Pathology, Peking University Shenzhen Hospital, Shenzhen, China; ^4^School of Traditional Chinese Medicine, Capital Medical University, Beijing, China; ^5^Department of Obstetrics and Gynecology, Guangdong Provincial People’s Hospital, Guangzhou, China; ^6^Peng Cheng Laboratory, Shenzhen, China

**Keywords:** digital pathology, immunohistochemistry, Ki-67, deep learning, fully convolutional network, neuroendocrine tumor

## Abstract

**Objective:**

To obtain molecular information in slides directly from H&E staining slides, which apparently display morphological information, to show that some differences in molecular level have already encoded in morphology.

**Methods:**

In this paper, we selected Ki-67-expression as the representative of molecular information. We proposed a method that can predict Ki-67 positive cells directly from H&E stained slides by a deep convolutional network model. To train this model, we constructed a dataset containing Ki-67 negative or positive cell images and background images. These images were all extracted from H&E stained WSIs and the Ki-67 expression was acquired from the corresponding IHC stained WSIs. The trained model was evaluated both on classification performance and the ability to quantify Ki-67 expression in H&E stained images.

**Results:**

The model achieved an average accuracy of 0.9371 in discrimination of Ki-67 negative cell images, positive cell images and background images. As for evaluation of quantification performance, the correlation coefficient between the quantification results of H&E stained images predicted by our model and that of IHC stained images obtained by color channel filtering is 0.80.

**Conclusion and Significance:**

Our study indicates that the deep learning model has a good performance both on prediction of Ki-67 positive cells and quantification of Ki-67 expression in cancer samples stained by H&E. More generally, this study shows that deep learning is a powerful tool in exploring the relationship between morphological information and molecular information.

**Availability and Implementation:**

The main program is available at https://github.com/liuyiqing2018/predict_Ki-67_from_HE

## Introduction

In recent years, deep learning has developed rapidly and has outperformed humans in some medical data analysis tasks (Li et al., 2018; Norgeot et al., 2019; von Chamier et al., 2019). Meanwhile, more and more tissue slides are digitalized by a scanner and saved as whole slide images (WSIs). Thus, it is natural to come up with the idea about applying deep learning algorithms to these WSIs. In fact, many researched tasks have explored the potential of deep learning on histopathological image analysis (Komura and Ishikawa, 2018), such as detection or segmentation of Region of Interest (ROI) (Spanhol et al., 2016), scoring of immunostaining (Mungle et al., 2017), mitosis detection (Roux et al., 2013) and so on.

In terms of pathology, hematoxylin and eosin (H&E), as the gold standard stain in evaluations for many cancer types, is routinely employed worldwide (Xu et al., 2019). In most cases, pathologists rely on H&E for their diagnosis and the majority of algorithms for histopathological image analysis, like cell detection, tissue segmentation and cancer grading, are based on H&E imaging (Ghaznavi et al., 2013). It is easy to acquire and cost effective. However, H&E stained slides only contain basic morphological information (Wittekind, 2003), such as the shapes of cells, tissues and tissue blocks. Molecular information like the expression of antigen (protein) in cells, which is more micro, is not reflected in H&E stained slides, which makes it difficult for pathologists and algorithms to analyze and assess.

To obtain molecular information in slides, immunohistochemistry staining (or IHC staining) is often employed in clinical practice. It allows the visualization of specific proteins on the tissue slide by binding targeted antibodies to corresponding proteins and highlighting the protein-binded antibodies by using chromogens of different colors (Ramos-Vara and Miller, 2014; Xu et al., 2019). Hence, this method can distinguish cells that express particular proteins from other components and therefore augment pathologist interpretation and direct therapy.

If a patient needs further diagnosis (such as confirming tumor subtype) or a targeted treatment plan, then an immunohistochemical test is often needed although he has already had H&E stained slides. It is because tumor subtype classification and making the plan of immunotherapy need some molecular information, which is not directly reflected in H&E staining slides. If this information can be inferred from H&E staining slides by some techniques like deep learning, it will greatly improve diagnostic efficiency and save costs.

If the assumption holds that the differences between positive cells (cells that contain a specific protein) and negative cells (cells that do not contain a specific protein) in IHC-stained slides have correlation with H&E-stained slides from the same regions, then there should be a way to model the relationship between the morphological information of cells in H&E images and IHC stained conditions of the cells. It is then possible to predict whether a cell can express specific proteins directly from a H&E-stained slide, without additional IHC staining process. In fact, some related works have been done to predict molecular information from H&E stained images. Coudray et al. (2018) founded six out of ten most commonly mutated genes in LUAD can be predicted from pathology images. Kather et al. (2019) showed that deep residual learning can predict microsatellite instability directly from H&E histology.

Ki-67 is a cancer antigen that is sometimes considered a good marker of proliferation, helping doctors determine patients’ cancer prognosis or their chance of recovery (Scholzen and Gerdes, 2000). However, in clinical practice, not every patient is tested for Ki-67 since it is time and money-consuming.

In this paper, we proposed a method that can predict Ki-67 positive cells directly from H&E stained slides by a deep convolutional network model, which realized a cell-level transformation. After the training process, the model was evaluated both on classification and quantification performance. The classification accuracies for our model on training set and validation set are 0.9780 and 0.9371. As for evaluation on quantification performance, the correlation coefficients of *D*_*pos*_, *D*_*neg*_ and *R*_*pos*_ between these two different types of images are 0.60, 0.73, and 0.80. The results reflect the consistency of Ki-67-expression between real IHC staining images and the output images of our model using H&E staining images as the inputs.

## Materials and Methods

**FIGURE 1:**
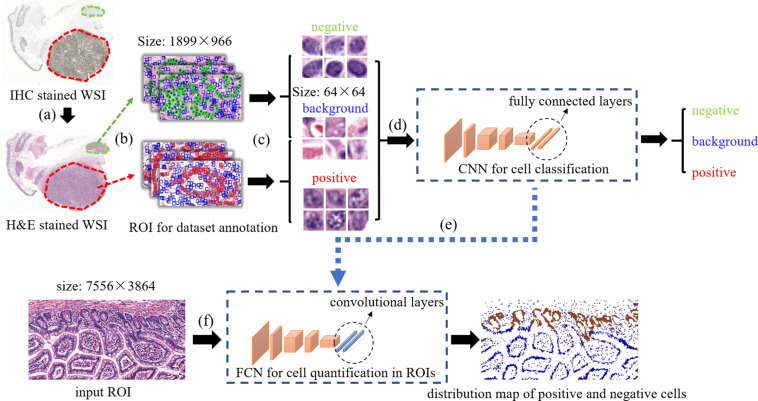
The overview of our method. **(a)** selecting positive/negative regions in H&E stained WSIs with the guidance of IHC stained WSIs, **(b)** ROIs selection for positive/negative/background samples extraction, **(c)** positive/negative/background samples annotation and extraction, **(d)** training CNN for cell classification, **(e)** transforming fully connected layers into convolutional layers, **(f)** taking a test ROI as the input of the transformed model for positive/negative cell distribution prediction.

The overview of our method is displayed on Figure 1. first, Consecutive sections of (formalin-fixed paraffin-embedded) samples obtained from the neuroendocrine tumor of twelve patients were cut and stained with H&E and Ki-67 antibody. Then, the slides were digitalized and a set of Ki-67 positive or negative cells in H&E stained images were annotated based on the Ki-67 expression present in the IHC stained images. After that, these cells along with some background patches were extracted for training the model. In order to quantify Ki-67 expressions in a bigger H&E stained image (sized 7,556 × 3,864 for each), a transformation was applied to our trained convolutional network to convert all the fully connected layers into convolutional layers. In this way, the transformed network can take one ROI as the input and output the classification map of the ROI. In order to compare real IHC staining images and images predicted by our model, we use color channel filtering to convert IHC staining images into three-value colormaps.

### Data Preparation

#### Patient Material

Formalin-fixed paraffin-embedded tumor samples of twelve patients operated for neuroendocrine tumor within the Peking university Shenzhen Hospital, China, were used in the study. The samples were stored in archives of Department of pathology in Peking university Shenzhen hospital and the Head of the Department of Pathology approved the use of the samples. The samples were anonymized and all patient-related data and unique identifiers were removed. The procedures were performed under the supervision and approval of the Ethics Committee in Peking university Shenzhen hospital. Samples represented different histological types: five cases with neuroendocrine tumor of rectum(G1), two cases of neuroendocrine tumor of colon(G3), one case of neuroendocrine tumor of small intestine(G1), two cases of neuroendocrine tumor of duodenum(G3), one case of gastric tubular adenocarcinoma with neuroendocrine tumor (G3) and one case of rectal tubular adenocarcinoma with neuroendocrine tumor (G3).

#### Staining Protocols

From each Formalin-fixed parafinembedded block, we cut two consecutive sections (3.5 μm): One for H&E staining and one for staining with the Anti-Ki67 antibody. For H&E staining, we used undiluted Mayer’s hematoxylin and 0.5% eosin. For IHC, we used Anti-Ki67 antibody (Roche, United States), 3,3’-diaminobenzidine as chromogen, and Mayer’s hematoxylin as a counterstain with a 1:10 dilution.

#### Sample Digitization

Matched H&E and IHC stained slides were scanned at 40× with Sqray slide scanner.

### Construction of the Dataset

Based on the Ki-67-expression, we selected 300 regions of interest (ROIs) sized 1,889 × 966 from 5 out of 12 H&E stained slides. Then we extracted 5,900 images of positive cells, 6,086 images of negative cells and 6,776 images of background from these ROIs.

**FIGURE 2:**
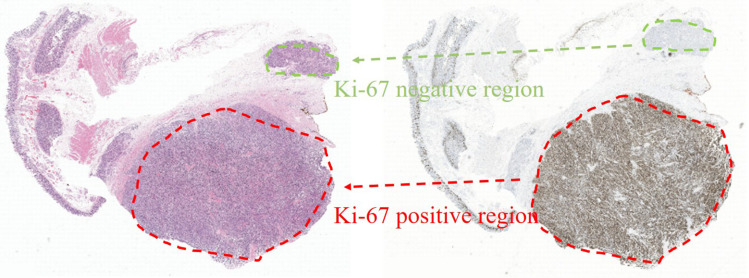
The way of selecting positive and negative samples.

The way of selecting positive and negative samples can be described with Figure 2. As is shown in Figure 2, there are Ki-67 positive regions in Ki-67 stained slides where all cells are Ki-67 positive. We can infer that the corresponding regions in H&E stained slides are also positive. Therefore, positive samples can be obtained by the following steps: First, extract images from the positive regions in H&E stained slides; Then, annotate each cell in these extracted images with a point label by using a open source annotation software Labelme. Finally, extract patches with these annotated points as centers and these patches are what we need. The way of obtaining negative samples is similar with that of obtaining positive samples.

**FIGURE 3:**
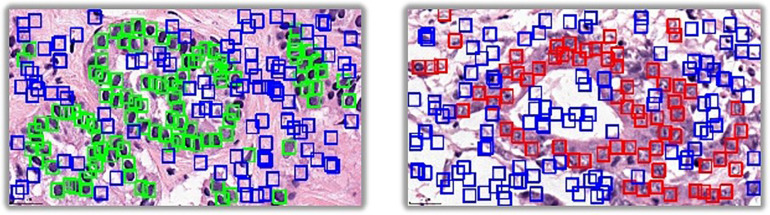
The method of extracting background samples.

The method of extracting background samples is shown in Figure 3. After the samples of positive cells and negative cells are selected, the background samples are selected by random sampling: a series of candidate boxes (shown in blue in Figure 3) are randomly generated. If the candidate boxes do not overlap with the boxes of negative cells (shown in green in Figure 3) or positive cells (shown in red in Figure 3), they will be retained and selected.

**FIGURE 4:**
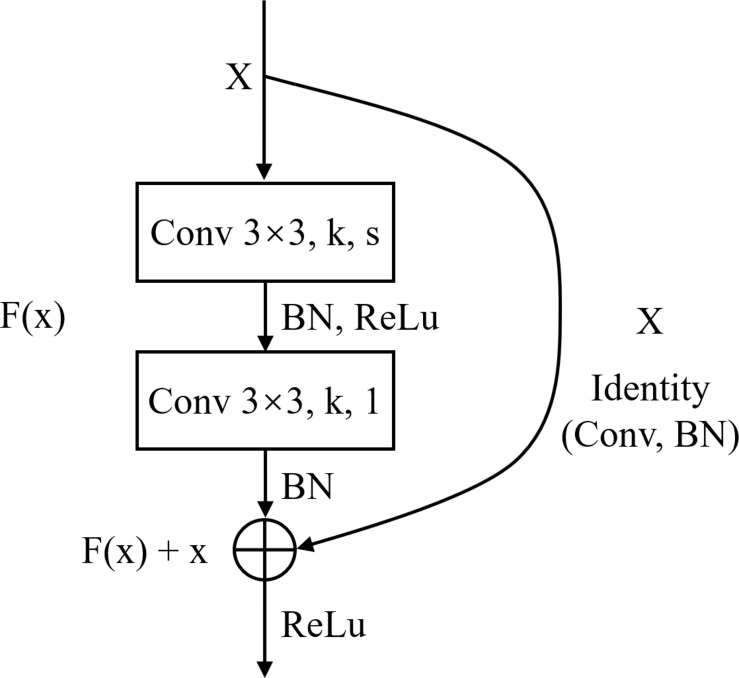
The structure of Block.

The size of these images was all 64 × 64 without any resize operation. The reason why we use 64 × 64 as the patch size is that the distribution of cell size is 40 pixel × 40 pixel ∼70 pixel × 70 pixel so the size of 64 × 64 can cover most situations. In addition, 64 is integer power of 2 which is convenient for computing. After that, the dataset consisting of all the images were split randomly into training set and validation set with the ratio of 8:2. The procedure of constructing the dataset were illustrated in Figures 1a–c. Table 1 summarizes information about the dataset.

**TABLE 1:** Information about the dataset.

**Parameters**	**Values**
Magnification/number of the H&E-IHC pairs	40×/12
Size/number of the ROIs selected for extracting training and validation set	1,889 × 966/300
Numbers of positive cell/negative cell background images	5,900/6,086/6,776
Ratio of training set to validation set	8:2
Size/number of the ROIs selected for evaluation of cell quantification	7,556 × 3,864/32

### Classification Using CNN

Deep Learning is a significant area of Machine Learning research. It uses very deep (in terms of number of layers) neural network to solve problems, especially problems which are related to visual recognition. The key aspect of all the deep learning architectures is the use of Convolutional Neural Network (CNN) (Krizhevsky et al., 2012). CNN is a biologically inspired form of the artificial neural network, that has local connections and shared weights. It is one of the most important tools of machine learning when it comes to the current generation, and it has been very popularly used to solve image recognition tasks, in the field of Computer Vision. The CNN architecture can be obtained by exploiting existing famous networks such as VGG (Simonyan and Zisserman, 2014), Inception (Szegedy et al., 2015) or ResNet (He et al., 2016), or by designing a new network. Both of them have their own strengths and weaknesses. Using existing networks can take advantages of pre-trained weights acquired from training the networks on large scale public datasets such as ImageNet (Deng et al., 2009) for transfer learning. This can accelerate the process of training significantly and guarantee the classification accuracy in the stage of inference, with only a small amount of training set. However, these pre-trained weights are usually generated by training the networks on natural images, which have considerable difference from histopathological images. So, the pre-trained models for natural image classification may not entirely appropriate for recognition tasks on histopathological images. Using self-designed network can be more flexible as we can devise a more targeted model according to the characteristics of the dataset. While this approach may not achieve a satisfied result if the training set is limited. ResNet is a well-known deep learning network architecture proposed by He et al. (2016). By using “shortcut connections,” this network are easier to optimize, and can gain accuracy from considerably increased depth. In this paper, we adopted a modified ResNet18 as our CNN classifier by removing the last average pooling layer to make the network adaptable for locating positive or negative cells in large-scale H&E stained images (which will be elaborated in the next subsection) and changing the number of the output nodes in the last fully connected layer into 3 since it was a 3-value classification problem. Table 2 lists detailed information about the modified ResNet18 network including layer name, input and output size, types of elements in each layer and their parameters. *k* represents the number of the kernels and *s* represents stride.

**TABLE 2:** Detailed information about the modified ResNet18 network.

**Layer name**	**Input size/output size**	**Elements/params**
conv1	3 × 64 × 64/64 × 32 × 32	Conv/7 × 7, *k* = 64, *s* = 2
		BN/-
		ReLu/-
pool1	64 × 32 × 32/64 × 16 × 16	MaxPool/3 × 3, *s* = 2
layer1	64 × 16 × 16/64 × 16 × 16	Block/*k* = 64, *s* = 1
		Block/*k* = 64, *s* = 1
layer2	64 × 16 × 16/128 × 8 × 8	Block/*k* = 128, *s* = 2
		Block/*k* = 128, *s* = 1
layer3	128 × 8 × 8/256 × 4 × 4	Block/*k* = 256, *s* = 2
		Block/*k* = 256, *s* = 1
layer4	256 × 4 × 4/512 × 2 × 2	Block/*k* = 512, *s* = 2
		Block/*k* = 512, *s* = 1
FC	512 × 2 × 2 (flattened)/3	fc/out = 3

*The structure of Block is shown in Figure 4.*

Figure 1d shows the stage of training CNN. The input to the first layer is an RGB image containing one positive or negative cell only or not containing any cell. The last layer generated labels, showing the probability of the image whether it represents a positive cell, a negative cell or background. Then a loss function was calculated and back propagation will be conducted to adjust the weighting parameters of the network so as to minimize the loss.

### Cell Quantification in ROIs Using Fully Convolutional Network

We had trained a CNN classifier using the samples of positive cell, negative cell and background. However, this classifier had a fixed size (64 × 64) of input and can only classify images with that size. In order to obtain the classification maps of ROIs (sized 7,556 × 3,864 for each), a transformation method proposed in Long et al. (2015) was applied to our trained CNN to convert all the fully connected layers into convolutional layers, as is shown in Figure 1e.

**FIGURE 5:**
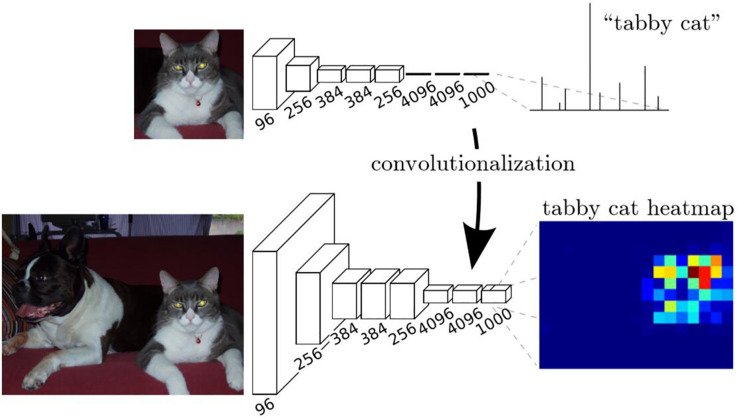
Description of the transformation method (Long et al., 2015).

The transformation method can be described in Figure 5. In training stage, the network learns a classification task. The input is a fixed size image, and the output is the corresponding category (cat for example) of the image. In the inference stage, the fully connected layer of the trained network is rearranged into a convolutional layer. In this way, the network can take any size of the image as input and output a probability map, representing the predicted probability of the target at each pixel in the input image.

Thus, the transformed CNN can take one ROI as the input and output the classification map of the ROI. The procedure was displayed in Figure 1f.

### Statistical Methods

To evaluate the classification result, we adopted accuracy, precision, recall, F1-score and confusion matrix. For illustration purposes, we will use T and F to indicate whether the network prediction is correct or not. P and N are used to indicate whether the sample is negative or positive. Therefore, TP (True Positive) means positive and correctly predicted, while FN (False Negative) means negative and wrongly predicted. The same is true for TN and FP. In this way, accuracy, precision and recall can be expressed as following:

$$\text{Accuracy}=\frac{T\,P+T\,N}{T\,P+F\,P+F\,N+T\,N}$$

$$\text{Precesion}=\frac{T\,P}{T\,P+F\,P}$$

$$\text{Recall}=\frac{T\,P}{T\,P+F\,N}$$

F1-score is defined as a harmonic mean of precision and recall:

$$F\,1=\frac{2\times \text{precision}\times \text{recall}}{\text{preicision}+\text{recall}}$$

Confusion matrix, which is represented by an n × n matrix, is a specific table layout that allows visualization of the performance of an algorithm. Each column of the matrix represents the instances in a predicted class while each row represents the instances in an actual class. A value in i column j row represent how many samples in class j is predicted to be class i.

To evaluate the quantification result, first we calculated dense of positive cells (*D*_pos_), proportional area of negative cells (*D*_neg_) and positive rate (*R*_pos_) in H&E ROIs and IHC ROIs respectively, which are defined as:

$$\left\{ \begin{array}{c} D_{\text{pos}}=\frac{S_{\text{pos}}}{S_{\text{ROI}}}\\ D_{\text{neg}}=\frac{S_{\text{neg}}}{S_{\text{ROI}}}\\ R_{\text{pos}}=\frac{S_{\text{pos}}}{S_{\text{pos}}+S_{\text{neg}}} \end{array}\right.$$

where *S*_*pos*_ is the area covered by positive cells, *S*_*neg*_ is the area covered by negative cells, *S*_*ROI*_ is the area of a whole ROI. The areas covered by positive cells or negative cells were obtained simply by color channel filtering. Then we computed pairwise correlation coefficient (*r*), as is defined in the equation below, for measuring correlation between the quantification results of H&E ROIs and that of IHC ROIs.

$$r\,\left(X,Y\right)=\frac{C\,o\,v\,\left(X,Y\right)}{\sqrt{V\,a\,r\,\left[X\right]\,V\,a\,r\,\left[Y\right]}}$$

where *cov(X, Y)* is defined as

C⁢o⁢v⁢(X,Y)=E⁢((X-E⁢(X))⁢(Y-E⁢(Y)))

## Experiments and Results

### Experimental Setup

In this section, we tested the performance of cell classification and cell quantification of our model. Table 3 shows the training details of our modified ResNet18 model.

**TABLE 3:** Training details of our modified ResNet18 model.

GPU	TITAN Xp (12GB) × 1
Framework	Pytorch 1.1.0
Data preprocessing	Random Horizontal Flip Random Vertical Flip Normalize (mean vector = [0.485, 0.456, 0.406], standard deviation vector = [0.229, 0.224, 0.225])
Batch size	64
Loss function	Cross Entropy
Learning rate	1e-3 (epoch 1–10), 1e-4 (epoch 11–20)
Optimizer	Adam with weight decay = 1e-5
Training on the pretrained model?	Yes (pretrained model from ImageNet.)
Training epochs	20

### Evaluation on Single Cell Classification

**FIGURE 6:**
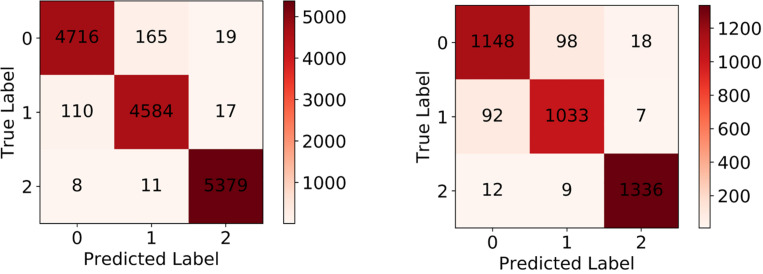
**Left:** Confusion matrix of training set. **Right:** Confusion matrix of validation set.

After the training process, we fed the validation set into our trained model to evaluate its classification performance. The classification accuracies for our model on training set and validation set are 0.9780 and 0.9371. Table 4 and Figure 6 left show the classification report and confusion matrix of the results of the train set respectively. Table 5 and Figure 6 right show those of the validation set. We also performed a 10-fold cross-validation analysis. We randomly split the training set (15,009 images) in to 10 subsets (nine sets of 1,501 images and one set of 1,500 images). In 10 training rounds, the average accuracy was 0.9310 (range: 0.9167–0.9427, std = 0.0085). These results are consistent with the previous results obtained from the validation set of 3,753 images, which suggest that the performance of our model is robust to how we split our dataset for training and test.

**TABLE 4:** Classification report on training set.

	**Precision**	**Recall**	**f1-score**	**Support**
Negative	0.962	0.976	0.969	4834
Positive	0.973	0.963	0.968	4760
Background	0.996	0.993	0.995	5415
Avg/total	0.978	0.978	0.978	15009

**TABLE 5:** Classification report on validation set.

	**Precision**	**Recall**	**f1-score**	**Support**
Negative	0.908	0.917	0.913	1252
Positive	0.913	0.906	0.909	1140
Background	0.985	0.982	0.983	1361
Avg/total	0.937	0.937	0.937	3753

### Evaluation on Cell Quantification in ROIs

**FIGURE 7:**
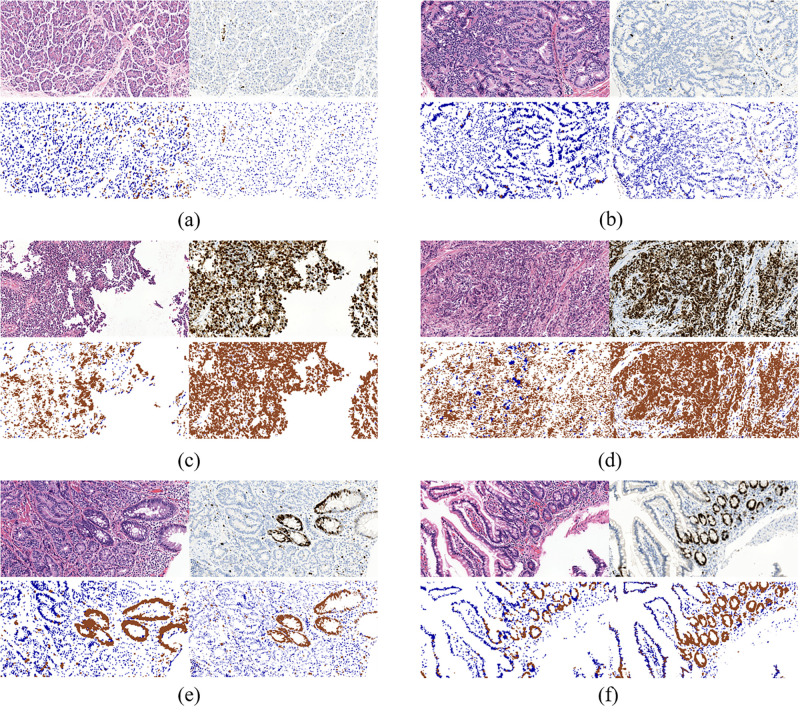
Three typical cases of Ki-67-expression: middle rate of Ki-67 positive cells **(a,b)**, low rate of Ki-67 positive cells **(c,d)** and high rate of Ki-67 positive cells **(e,f)**. Each case contains four sub-figures, representing H&E stained ROIs (top left), IHC stained ROIs (top right), quantification results in H&E stained ROIs (bottom left) and quantification results in IHC stained ROIs (bottom right).

To further evaluate our model, we compared the quantification results in H&E stained ROIs performed by the model and the results in IHC stained ROIs performed by color channel filtering. Figure 7 displays three typical cases of Ki-67 expression. It’s obvious that the quantification results in H&E stained ROIs are basically consistent with the quantification results in IHC stained ROIs.

Specifically, the results in middle rate cases and low rate cases are better than those in high rate cases. It is because in ROIs with high rate of Ki-67 positive cells, the distribution of positive and negative cells is more irregular than the other two types of cases, which makes it difficult to distinguish positive cells from negative cells in H&E stained ROIs in the process of annotation. It is because when we label the cells, due to the lack of correspondence between scattered cells in the H&E stained regions and Ki-67 stained regions, it was not completely determined whether a certain cell was a positive cell or a negative cell in H&E stained ROIs unless all the cells in these regions are all positive or negative. Though there are a mass of positive cells in Ki-67 positive regions, a small number of negative cells are inevitably mixed in with positive cells in this type of ROIs, which makes labeling more difficult. While the similar situations appear less in Ki-67 negative regions. In other words, negative cells in Ki-67 negative regions can be extracted with more confidence than positive cells in Ki-67 positive regions.

In addition, there are many glandular-like structures in ROIs with low or medium density of negative cells. If all the cells on a gland in KI-67 stained ROIs are negative or positive, then all the cells in the corresponding gland in H&E stained ROIs are also marked as negative or positive, according to the correspondence between glands in H&E stained ROIs and Ki-67 stained ROIs.

**FIGURE 8:**
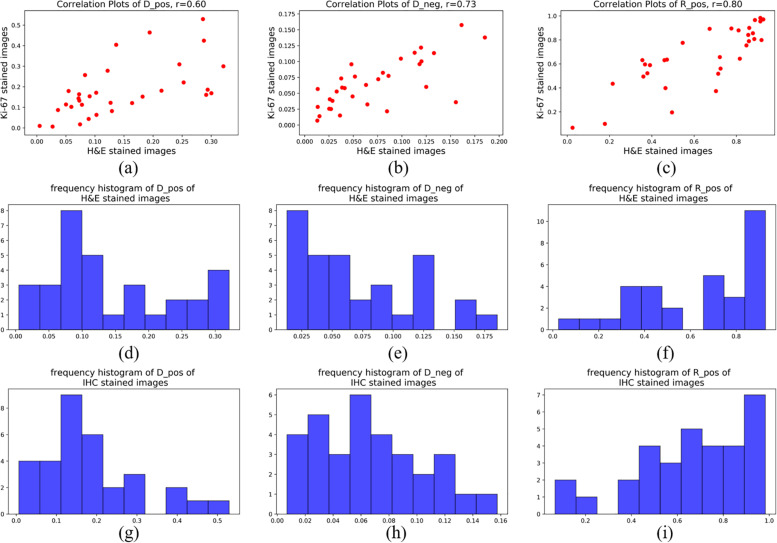
Frequency histogram of **(a)** Dpos, **(b)** Dneg and **(c)** Rpos of H&E stained images; Frequency histogram of **(d)** Dpos, **(e)** Dneg and **(f)** Rpos of IHC stained images; Correlation-plots of H&E stained images and IHC stained images of **(g)** Dpos, **(h)** Dneg and **(i)** Rpos.

For statistical evaluation, we calculated *D*_*pos*_, *D*_*neg*_ and *R*_*pos*_ in 32 pairs of H&E-staining ROIs and IHC-staining ROIs. Figure 8 shows the frequency histograms and correlation plots of these three indexes in H&E stained images and IHC stained images. The correlation coefficients of *D*_*pos*_, *D*_*neg*_ and *R*_*pos*_ between these two different types of ROIs are 0.60, 0.73 and 0.80. The results reflect the quantitative consistency of Ki-67 expression between the two types of staining images. Moreover, The correlation coefficients of *R*_*pos*_ has the highest value indicates that the evaluation indexes considering both positive and negative cells can more stablely reflect the relationship between H&E stained ROIs and Ki-67 stained ROIs.

## Discussion

In this paper, we made an attempt to build a relationship between H&E stained slides and Ki-67 antibody stained slides. We introduced a modified ResNet18 model to predict Ki-67 expression directly from H&E stained images without any IHC staining process. Our results show that morphological information has close relation with molecular information, which are consistent with the opinion proposed in Fuchs and Buhmann (2011) that tissue and cell morphologies displayed in histopathological images are a function of underlying molecular drivers. Once their relationship is established, it should be possible to faithfully predict the distribution of specific protein abundance directly in samples only using a basic morphology staining. However, it’s just the beginning of our research on this topic. Challenges still exist, including:

1. Performance of the model is highly dependent on the quality of input images. Low quality images may result in less accurate results. The quality of images is influenced by many factors, such as standardization of making slides, quality of stains and accuracy of scanners.
2. The relationship between morphological information and molecular information may be very complex, considering the diversity of different lesions, tissues, cells and antibodies. For the moment, our research has only focused on one specific relationship so much work should be done if we want our model to be more generalized.
3. At present we can only distinguish between positive cells and negative cells in some certain regions of a H&E stained images guided by the corresponding IHC stained image. It’s hard to verify the positive degree of a cell in a H&E stained image even with the help of IHC staining, which hampers a more precise inference of the model.

Our future work will mainly focus on the following aspects. first, Enlarge our dataset to contain more samples. So, the model trained on the new dataset will have stronger ability of robust and generalization; Second, Conduct more experiments on samples with different tissues and stains to promote our conclusion to a more general situation; Last but not least, Optimize our model. For example, semi-supervised learning can be adopted to alleviate the workload of annotation.

## Data Availability Statement

The raw data supporting the conclusions of this article will be made available by the authors, without undue reservation.

## Author Contributions

YH presented the initial idea for the article and provided financial support. YL wrote all the code, conducted experiments and wrote most of the article. XL and AZ communicated with the hospital and obtained access to the data. They also provided guidance on pathological diagnosis, verified the medical significance of our work, and wrote part of the article. YC provided support on computing resources and guidance on deep learning techniques. XZ and ML reviewed the data and the results of the experiment and gave valuable suggestions for the revision of the article. QL and HL made and collected all the slides. SL scanned all the slides and proposed the method of data labeling. MH finished the work of data labeling. All authors contributed to the article and approved the submitted version.

## Conflict of Interest

The authors declare that the research was conducted in the absence of any commercial or financial relationships that could be construed as a potential conflict of interest.
